# Synthesis of ^15^N‐labelled 3,5‐dimethylpyridine

**DOI:** 10.1002/jlcr.3807

**Published:** 2019-11-14

**Authors:** Mario Schubert, Hans‐Heinrich Limbach, José Elguero

**Affiliations:** ^1^ Institute of Chemistry and Biochemistry Free University of Berlin Berlin Germany; ^2^ Instituto de Química Médica (IQM) Consejo Superior de Investigaciones Científicas (CSIC) Madrid Spain; ^3^ Institute of Molecular Biology University of Salzburg Salzburg Austria

**Keywords:** isotope labelling, lutidine, nitrogen‐15, pyridine, , 2*H*‐pyran, nitrogen‐15, isotope labelling, synthesis, 2*H*‐pyran

## Abstract

^15^N‐labelled pyridines are liquid‐ and solid‐state nuclear magnetic resonance (NMR) probes for chemical and biological environments because their ^15^N chemical shifts are sensitive to hydrogen‐bond and protonation states. By variation of the type and number of substituents, different target pyridines can be synthesized exhibiting different p*K*_a_ values and molecular volumes. Various synthetic routes have been described in the literature, starting from different precursors or modification of other ^15^N‐labelled pyridines. In this work, we have explored the synthesis of ^15^N ^15^N‐labelled pyridines using a two‐step process via the synthesis of alkoxy‐3,4‐dihydro‐2*H*‐pyran as precursor exhibiting already the desired pyridine substitution pattern. As an example, we have synthesized 3,5‐dimethylpyridine‐^15^N (lutidine‐^15^N) as demonstrated by ^15^N‐NMR spectroscopy. That synthesis starts from methacrolein, propenyl ether, and ^15^N‐labelled NH_4_Cl as nitrogen source.

## Graphical abstract

The synthesis of ^15^N labelled 3,5‐dimethylpyridine is achieved in two steps starting from methacrolein, 1‐propenyl ether and ^15^NH_4_Cl.

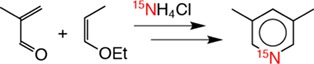

## 1 INTRODUCTION

^15^N‐labelled pyridines and related heterocycles are important liquid‐ and solid‐state nuclear magnetic resonance (NMR) probes for chemical and biological environments.^1^, ^2^, ^3^, ^4^, ^5^, ^6^ That feature arises on one hand from the basicity of pyridines and their ability to form hydrogen bonds. On the other hand, ^15^N chemical shifts are very sensitive to the ^15^N‐^1^H distance and can be used to monitor the local H‐bond and protonation state.^7^, ^8^, ^9^ Therefore, ^15^N‐labelled pyridines have been used to explore the acidity of mesoporous surfaces or of biological environments using high‐resolution solid‐state NMR spectroscopy. Moreover, the mobility of pyridines to jump from one proton donor to another allows one to obtain interesting information about local structures.^2^

**Scheme 1:**
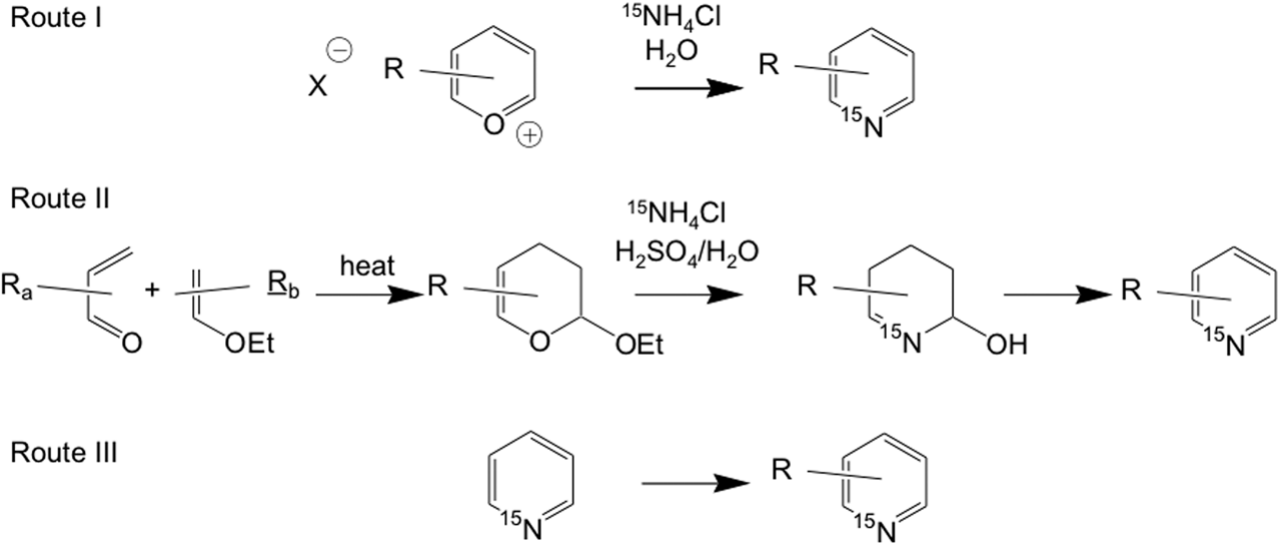
Synthetic routes to ^15^N‐labelled pyridine derivatives

**Scheme 2:**
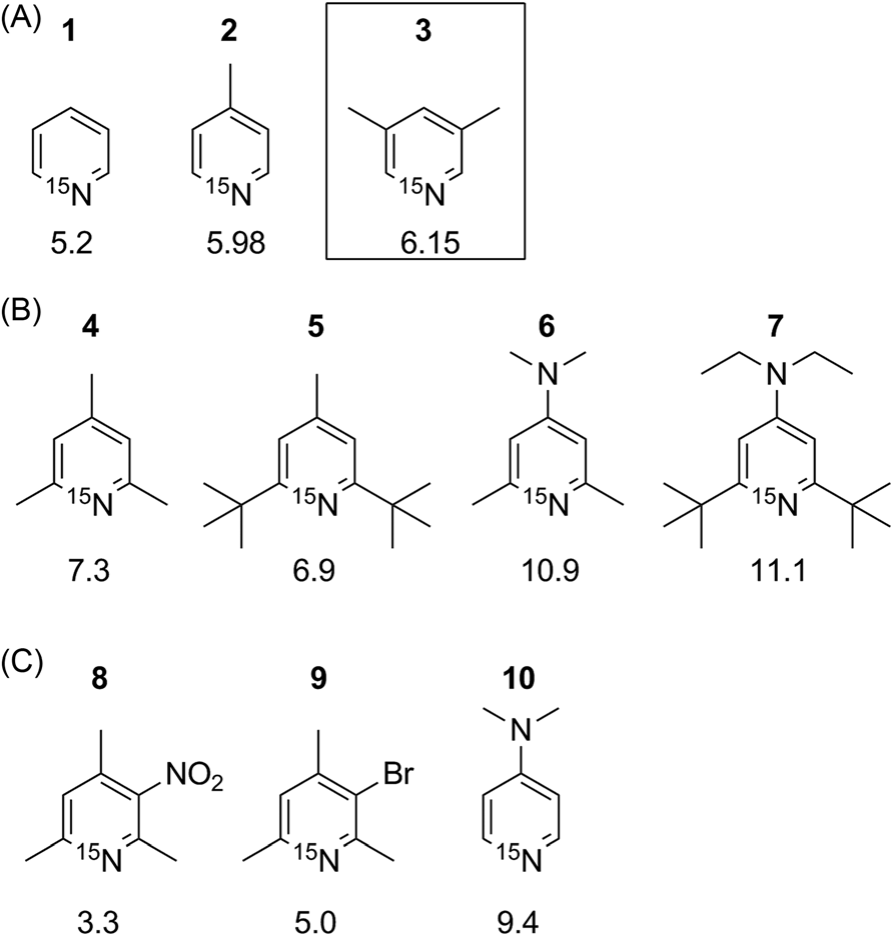
Overview of the so‐far reported ^15^N‐labelled pyridine derivatives obtained either by Route II (A), Route I (B), or Route III (C)

So far, ^15^N pyridine is the only representative that is commercially available in a ^15^N‐labelled form. Thus, syntheses of various ^15^N‐labelled pyridine derivatives with a large range of pKa values have been reported so far, applying several routes as illustrated in Scheme 1. Route I starts from the appropriate pyrylium salt containing already the desired pyridine substituents, using ^15^NH_4_Cl as nitrogen source. In Route II, alkoxy‐3,4‐dihydro‐2*H*‐pyrans exhibiting the desired substituents are firstly synthesized as precursors via a Diels‐Alder addition of vinyl ethers to α,β‐unsaturated carbonyl compounds. The pyrans can then easily be converted into the corresponding ^15^N‐labelled pyridines using ^15^NH_4_Cl. Finally, easily available labelled pyridines can be converted into other derivatives (Route III). Some examples are depicted in Scheme 2.

Up to date, most ^15^N‐labelled pyridines have been synthesized following Route I, namely, 2,4.6‐trimethyl‐pyridine **4,** also called collidine,^10^ 2,6‐di‐tert‐butyl‐4‐methyl‐pyridine **5**,^11^ 4‐dimethylamino‐2,6‐dimethy‐pyridine **6,** 12 and 4‐diethylamino‐2,6‐di‐tert‐butyl‐pyridine **7**.^11^ However, Route I is limited to pyridines with aliphatic substitutions at C2 and C6. Route II has been used to synthesize ^15^N‐labelelled plain pyridine **1** 12, 13 and 4‐methyl‐pyridine **2**.^9^ Route III was used for the synthesis of ^15^N‐labelled 2,4.6‐trimethyl‐3‐nitro‐pyridine **8**, 2,4.6‐trimethyl‐3‐bromo‐pyridine**9** and 4‐N,N‐dimethylamino‐pyridine **10**.^12^

As we wanted to obtain 3,5‐dimethyl‐pyridine‐^15^N (**3**) as molecular sensor for comparison with pyridine‐^15^N (**1**) and collidine‐^15^N (**4**), we explored the most suitable route to synthesize **3**. We could not use Route I as pyrylium salts without substituents in 2‐ and 6‐ position are rare and not very stable.^14^ In addition, it is not possible to obtain **3** from **1** via Route III. Therefore, we checked in more detail Route II. That route had been used to synthesize pyridine‐^15^N (**1**). The required precursor 3,4‐dihydro‐2‐methoxy‐2*H*‐pyran is commercially available and can be synthesized in solution at high pressures up to 15 000 bar^15^ or under milder conditions using either dry‐state adsorption conditions^16^ or an ytterbium catalyst.^17^ The original synthesis of Longley and Emerson^18^ did not use a solvent or additives but only the neat reactants, heating them up to about 200 °C in a normal laboratory autoclave. The pressure achieved was not reported, but they probably did not exceed about 15 bar.^18^ Therefore, that method seemed to us preferable as only small quantities of the pyran are needed. We found that this method was suitable and succeeded to synthesize in a similar way also 4‐methyl‐pyridine‐^15^N (**2**).^9^Therefore, we want to describe here in more detail how to prepare pyridines for which commercial precursors are not available, using the example of ^15^N‐labelled 3,5‐dimethylpyridine (**3**).

## 2 RESULTS AND DISCUSSION

In the first stage of this work, we checked out alternative routes starting from unlabelled 3,5‐dimethyl pyridine, but these efforts were not successful.

**Scheme 3:**
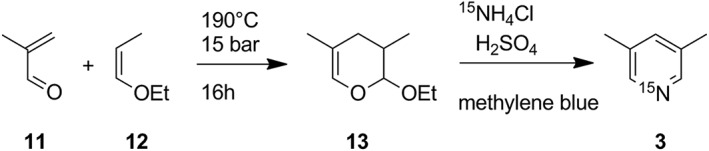
Synthetic route to ^15^N‐labelled lutidine used in this work

**Figure 1:**
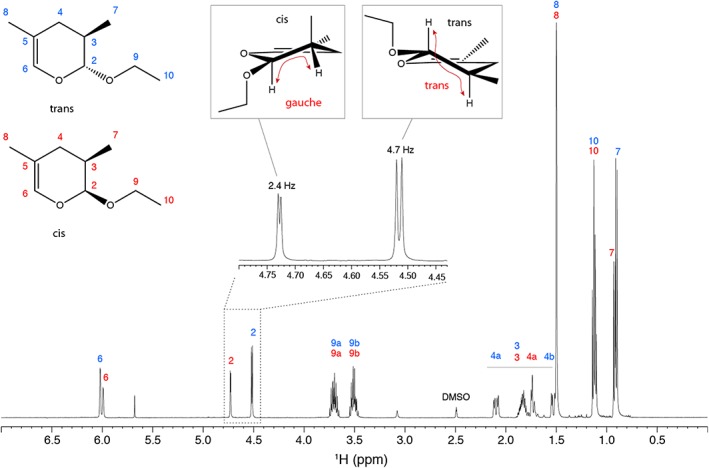
^1^H NMR spectrum of 2‐ethoxy‐3,4‐dihydro‐3,5‐dimethyl‐2*H*‐pyran **13** consisting of a 2:3 *cis*/*trans* mixture measured in DMSO‐*d*_*6*_. For clarity, only one enantiomer is shown for each diastereomer (2*R*,3*R* for *trans* and 2*S*,3*R* for *cis*). The dominating trans form shows a larger ^3^ *J*_H2H3_ scalar coupling. Signals between 1.5 and 2.2 ppm were only tentatively assigned

As precursor of the Diels‐Alder reaction, we used methacrolein **11** and ethyl 1‐propenyl ether **12** leading to 2‐ethoxy‐3,4‐dihydro‐3,5‐dimethyl‐2*H*‐pyran **13** (Scheme 3). Methacrolein was stabilized with a small amount of hydroquinone to avoid polymerization. Nuclear magnetic resonance spectroscopy revealed a *cis/trans* mixture of compound **13** in the ratio of 2:3 (Figure 1); the chemical shifts are listed and compared to literature values in Table 1. The chemical shifts and coupling constants of the two 2*H*‐pyran ring isomers fit very well to previous reports for 2‐benzoyloxy‐3,4‐dihydro‐3,5‐dimethyl‐2*H*‐pyran from Yamamoto et al^19^ and 2‐methoxy‐3,4‐dihydro‐3,5‐dimethyl‐2*H*‐pyran from Descotes et al.^20^ Although the diastereomers could potentially be separated by chromatography, a separation was not required because both diastereomers are an in situ source of 1,5‐pentane‐dial that is generated in the initial part of the second step of the synthesis. A side product of the reaction was the Diels‐Alder reaction of methacrolein with itself forming 3,4‐dihydro‐2*H*‐pyran‐2‐carbaldehyde, which was however reduced by using an excess of the dienophile and was separated by distillation.

**Scheme 4:**
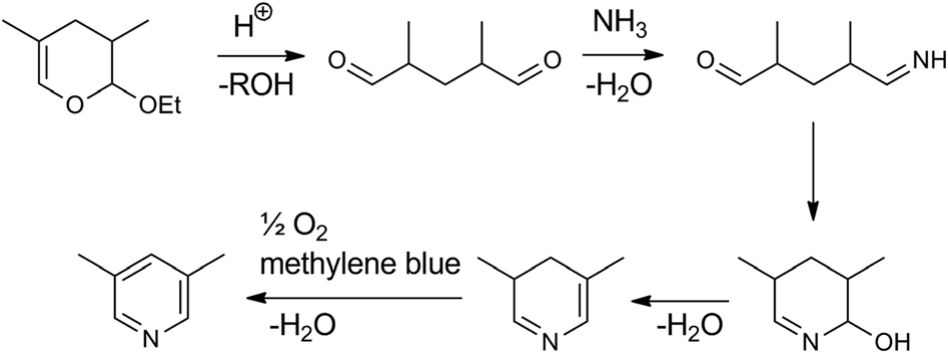
Proposed mechanism of the formation of 3,5‐dimethylpyridine in analogy to Whaley and Ott^13^

In the second step, the dihydropyran mixture was converted to 3,5‐dimethylpyridine according to Scheme 4. ^15^N‐labelled 3,5‐dimethylpyridine was isolated as an aqueous azeotrope by steam distillation of the basified reaction mixture, after volatile substances were initially removed by distillation of the acidic reaction mixture. Methylene chloride was used to extract the product from the azeotrope with yields of ~ 55% relative to the amount of the ^15^N isotope used.

### 2.1 NMR characterization of 3,5‐dimethylpyridine

The ^1^H NMR spectrum of ^15^N‐labelled 3,5‐dimethylpyridine was identical to the unlabelled compound, except that the protons adjacent to the ^15^N nucleus show a splitting of 10.6 Hz due to the ^2^ *J*_HN_ scalar coupling. The coupling is in agreement with previous reports measured in the same solvent.^21^ The observed ^1^H and ^13^C resonances agree with previously reported values measured in the same solvent,^22^ except that we assigned the ^13^C signal at 137.0 ppm to C3/C5 and 132.4 ppm to C4 (swapped in Pazderski et al). Our ^13^C assignment agrees also with the data measured in DMSO‐*d*_*6*_ and D_2_O despite small deviations due to the different solvents.^23^, ^24^ Although the ^13^C spectrum of labelled and unlabelled 3,5‐dimethylpyridine looked virtually identical, a closer interpretation revealed a small splitting of two signals due to small ^n^ *J*_CN_ scalar couplings. Interestingly, no splitting was observed for the two carbons directly adjacent to the nitrogen nucleus, but the signals of C_3_, C_5,_ and C_4_ showed a splitting. Values of 3.1 Hz for ^2^ *J*_CN_ and of 3.5 Hz for ^3^ *J*_CN_ were observed, which are in a similar range as those observed in ^15^N‐labelled pyridine (^*2*^ *J*_*CN*_ = –2.53 Hz and ^3^ *J*_CN_ = –3.85 Hz 25). ^1^ *J*_CN_ was too small to be detectable in a splitting in agreement with a ^1^ *J*_CN_ of 0.67 Hz observed for ^15^N‐labelled pyridine.^26^ The observed ^15^N resonance of –69.7 ppm referenced to CH_3_NO_2_ agrees well with previously reported chemical shifts measured at natural abundance.^21^, ^22^ A comparison with ^15^N chemical shifts of other methyl‐substituted pyridine derivatives is given in Supplementary Table S1.

**Table 1:** Chemical shifts of the mixture of 2‐ethoxy‐3,4‐dihydro‐3,5‐dimethyl‐2*H*‐pyran diastereomers and comparison with values of *cis* and *trans* 2‐benzoyloxy‐3,4‐dihydro‐3,5‐dimethyl‐2*H*‐pyran from Yamamoto et al^19^ and 2‐methoxy‐3,4‐dihydro‐3,5‐dimethyl‐2*H*‐pyran from Descotes et al^20^

Atom	Observed (DMSO‐*d*_*6*_) trans	Observed (DMSO‐*d*_*6*_) cis	Yamamoto (CDCl_3_) trans	Yamamoto (CDCl_3_) cis	Descotes (CCl_4_) trans	Descotes (CDCl_4_) cis
H2	4.52, d, *J* = 4.6 Hz	4.73, d, *J* = 2.3 Hz	4.60, d, *J* = 4.0 Hz	4.79, d, *J* = 2.1 Hz	4.35, d, *J* = 3.4 Hz	4.52, d, *J* = 1.7 Hz
H3	1.5‐2.2 ov ^a^	1.5‐2.2 ov ^a^	1.98 dddt	1.90 m		
H41	1.5‐2.2 ov ^a^	1.5‐2.2 ov ^a^	2.24 dd	1.76 m		
H42	1.5‐2.2 ov ^a^	1.5‐2.2 ov ^a^	1.53 dd	0.96 m		
H6	6.02, d, *J* = 1.4 Hz	5.99, d, *J* = 1.4 Hz	6.03, d, *J* = 1.2 Hz	6.02, d, *J* = 1.2 Hz	5.91	5.91
H7	0.90, d, *J* = 6.9 Hz	0.92, d, *J* = 6.5 Hz	0.96, d, *J* = 7.0 Hz	1.00, d, *J* = 6.4 Hz	0.91‐1.51	0.96‐1.52
H8	1.50 ov	1.50 ov	1.54, d, *J* = 1.2 Hz	1.55, d, *J* = 1.2 Hz	0.91‐1.51	0.96‐1.52
H1’/H1”	3.50‐3.70 ov	3.50‐3.70 ov	4.82 d, 4.57 d	4.78 d, 4.55 d	3.32	3.33
H2’	1.13 ov	1.13 ov	‐	‐		
C2	100.1	97.9	99.8	97.8		
C3	29.9 ^b^	30.5	30.3	31.0		
C4	29.8 ^b^	29.4	30.2	29.7		
C5	107.0	108.1	108.2	109.7		
C6	134.1	134.0	134.0	133.8		
C7	16.2	15.8	16.5	16.2		
C8	18.0	17.9	18.4	18.3		
C1’	62.9	62.9	69.4	69.1		
C2’	15.0	14.9				

Individual assignment could not be achieved because of overlapping signals (ov: overlap).

Assignment might be swapped.

In addition to NMR spectroscopy, mass spectrometry confirmed the chemical identity of compound **3**, whose mass spectrum differed from the unlabelled 3,5‐dimethylpyridine,^27^ only for the ^15^N‐containing fragments.

## 3 CONCLUSION

^15^N‐labelled 3,5‐dimethylpyridine could be conveniently synthesized in two steps starting from methacrolein, 1‐ethoxypropene, and ^15^NH_4_Cl.

## 4 EXPERIMENTAL

Unlabelled reagents were purchased from Sigma‐Aldrich. ^15^N‐labelled NH_4_Cl was purchased from Chemotrade Chemiehandelsgesellschaft (Leipzig, Germany).

### 4.1 NMR spectroscopy and mass spectrometry

Unless stated otherwise, NMR spectra were recorded either on a Bruker AMX 500 or a Bruker AMSY 270 with CDCl_3_ as solvent at 298K. ^1^H and ^13^C chemical shifts were referenced to TMS. The solvent signals of signals were set for DMSO‐*d*_*6*_ to 2.49 ppm (^1^H) and 39.51 ppm (^13^C) and for CDCl_3_ to 7.24 ppm (^1^H) and 77.2 ppm (^13^C). ^15^N resonances were indirectly referenced to CH_3_NO_2_, using a saturated solution of ^15^NH_4_Cl in H_2_O (~5.64 M) with a chemical shift of –352.89 ppm.^28^ Mass spectra were recorded on a Varian MAT 711.

### 4.2 Synthesis of 2‐ethoxy‐3,4‐dihydro‐3,5‐dimethyl‐2*H*‐pyran 13

14 g (0.2 mol) of methacrolein **11**, 26 g (0.3 mol) of 1‐ethoxypropene **12,** and 0.1 g of hydroquinone (0.25% of mixture) were heated in a 200‐ml autoclave (high‐pressure laboratory autoclave model II from Carl Roth, Germany) at 190 °C for 16 hours. During that time, the pressure first rose to 15 bar and then fell to 8 bar. After cooling, the reaction mixture was distilled under reduced pressure of 48 mbar, yielding at 97°C 18.8 g of a fruity‐smelling colorless oil. The product **13** was further purified by column chromatography (Al_2_O_3_, hexane/ethyl acetate 10:1, column dimensions 40 × 6 cm). Yield: 11.7 g (74.8 mmol; 37%). n_D_ 20 = 1.4420. TLC (Al_2_O_3_ hexane/ethyl acetate 10:1): R_f_ = 0.727. ^1^H‐NMR (DMSO‐*d*_*6*_): 6.02 (s, 0.59H, H6trans), 5.99 (s, 0.41H, H6cis), 4.73 (d, 0.41H, H2cis, *J* = 2.3 Hz), 4.52 (d, 0.59H, H2trans, *J* = 4.6 Hz), 3.5‐3.7 (m, 2H, CH_2_CH_3_), 1.5‐2.6 (m, 3H, H3/H4), 1.5 (s, 3H, 5‐CH_3_), 1.12 (q, 3H, CH_2_ CH_3_), 0.91 (2×d, 3H, 3‐CH_3_). ^13^C‐NMR (DMSO‐*d*_*6*_): 134.1 (C6), 108.1 (C5cis), 107.0 (C5trans), 100.0 (C2trans), 97.9 (C2cis), 62.9 (CH_2_CH_3_), 29.4‐30.4 (C3 and C4), 17.9 (5‐CH_3_), 16.2 (3‐CH_3_ trans), 15.7 (3‐CH_3_ cis), 15.0 CH_2_ CH_3_). MS (EI): 156 (18, M^+^), 111 (19), 86 (100, retro‐Diels‐Alder), 58 (90).

### 4.3 Synthesis of 3,5‐dimethylpyridine 3

In a three‐necked flask equipped with a reflux condenser, an addition funnel, and a magnetic stirrer, 150 ml of deionized water were poured, followed by 4.4 ml of concentrated H_2_SO_4_, 15 g (39.7 mmol) of methylene blue, and 2 g (36.7 mmol) of ^15^NH_4_Cl. The solution was brought to reflux, and a solution of 5.78 g (37 mmol) of 2‐ethoxy‐dihydro‐3,5‐dimethyl‐2H‐pyran **13** in 5 ml of ethanol was added dropwise over a period of 1 hour and refluxed for 17 hours. After cooling, 150 ml of deionized water was added and the mixture was distilled until the odor of glutaraldehyde disappeared in the distillate (ca. 200 ml). After cooling of the remaining reaction mixture, 250 ml of 1.3 M NaOH were added gradually and distilled until ~200 ml of distillate was collected. 0.1 g of Na_2_CO_3_ was added, and CH_2_Cl_2_ was used to extract the organic base. The combined organic layers were dried with Na_2_SO_4,_ and the solvent was removed with a rotary evaporator. Yield: 2.18 g (20.2 mmol; 55%). ^1^H‐NMR (CDCl_3_): 8.21 (d, 2H, H2/H6, ^2^ *J*_NH_ = 10.6 Hz), 7.26 (s, 1H, H4), 2.25 (s, 6H, CH_3_), ^13^C‐NMR (CDCl_3_): 147.3 (C2/C6), 137.0 (d, ^3^ *J*_NC_ = 3.5 Hz, C4), 132.4 (d, ^2^ *J*_NC_ = 3.1 Hz, C3/C5), 18.1 (CH_3_). ^15^N‐NMR (CDCl_3_): –69.7 ppm referenced to CH_3_NO_2_. MS (EI): 108 (100, C_7_H_9_^15^N^+^), 93 (21), 79 (35), 77 (11).

## Supporting information


**Table S1** Comparison of 15N chemical shifts of pyridine derivatives measured in CDCl3 at 298 K and referenced to external CH3NO2
