# Supplementary material for: Synthesis of 15N‐labelled 3,5‐dimethylpyridine
Source: J Labelled Comp Radiopharm. 2019 Nov 14;62(14):914–9. doi: 10.1002/jlcr.3807 (PMC6973028; doi:10.1002/jlcr.3807)
Supplement: Supplementary file 1 — Table S1 Comparison of 15N chemical shifts of pyridine derivatives measured in CDCl3 at 298 K and referenced to external CH3NO2 [file JLCR-62-914-s001.pdf]

SUPPLEMENTARY MATERIAL

Synthesis of ^{15}N labelled 3,5-dimethylpyridine

Mario Schubert, Hans-Heinrich Limbach, José Elguero

Content:

Supplementary Table 1	page 2
^{1}H spectrum of 2-ethoxy-3,4-dihydro-3,5-dimethyl-2*H*-pyrane **13**	page 3
^{13}C spectrum of 2-ethoxy-3,4-dihydro-3,5-dimethyl-2*H*-pyrane **13**	page 4
^{1}H spectrum of 3,5-dimethylpyridine **3**	page 5
^{13}C spectrum of 3,5-dimethylpyridine **3**	page 6
^{15}N spectrum of 3,5-dimethylpyridine **3**	page 7

Supplementary Table 1: Comparison of ^{15}N chemical shifts of pyridine derivatives measured in $CDCl_3$ at 298 K and referenced to external CH_3NO_2.

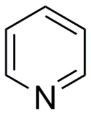	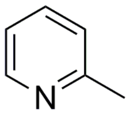	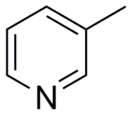	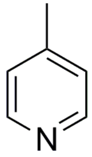	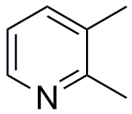	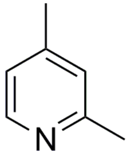	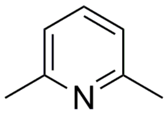	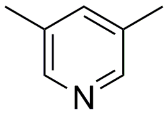	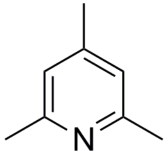	
pyridine	2-picoline	3-picoline	4-picoline	2,3-lutidine	2,4-lutidine	2,6-lutidine	3,5-lutidine	2,4,6-collidine	References
							-69.7 ppm		This work
							-69.8 ppm[a]		(1)
-70.1 ppm[b]	-71.6 ppm[b]	-75.8 ppm[b]	-84.1 ppm[b]			-72.2 ppm[b]			(2)
				-71.4 ppm[c]	-80.0 ppm[c]	-72.0 ppm[c]	-71.0 ppm[c]	-80.1 ppm[c]	(3)
-67.7 ppm[d]	-68.8 ppm[d]	-68.4 ppm[d]	-76.7 ppm[d]						(4)

[a] ^{15}N measurements at natural abundance in different solvents, among them $CDCl_3$; data were measured at 302 K and referenced to external CH_3NO_2

[b] Dodalik et al. reported ^{15}N data in $CDCl_3$ as well as DMSO-d_6 at 300K, at natural abundance, referenced to external liquid NH_3; for this table we calculated the chemical shifts referenced to external CH_3NO_2 by subtracting 381.7 ppm according to Marek and Lycka 2002 (5) and Pazderski et al. 2009 (6).

[c] data measured with $CDCl_3$ at 303 K, at natural abundance, referenced to external CH_3NO_2

[d] data measured at with $CDCl_3$ at 298 K, at natural abundance, referenced to external CH_3NO_2

References:

1. Holzer W & von Philipsborn W (1989) Inter-Molecular and Intra-Molecular Hydrogen-Bonding Effects on Geminal N-15, H-1 Spin Coupling and N-15 Chemical-Shifts in Pyridine-Derivatives. *Magn Reson Chem* 27(6):511-514.
2. Dokalik A, Kalchhauser H, Mikenda W, & Schweng G (1999) NMR spectra of nitrogen-containing compounds. Correlations between experimental and GIAO calculated data. *Magn Reson Chem* 37(12):895-902.
3. Pazderski L, Pawlak T, Sitkowski J, Kozerski L, & Szlyk E (2010) Structural correlations for H-1, C-13 and N-15 NMR coordination shifts in Au(III), Pd(II) and Pt(II) chloride complexes with lutidines and collidine. *Magn Reson Chem* 48(6):417-426.
4. Jazwinski J & Duddeck H (2003) Pyridine and aminide derivatives as ligands in 1 : 1 Rh-2[tfa](4) adducts: H-1, C-13 and N-15 NMR study. *Magn Reson Chem* 41(11):921-926.
5. Marek R & Lycka A (2002) N-15 NMR spectroscopy in structural analysis. *Curr Org Chem* 6(1):35-66.
6. Pazderski L*, et al.* (2009) Experimental and quantum-chemical studies of H-1, C-13 and N-15 NMR coordination shifts in Au(III), Pd(II) and Pt(II) chloride complexes with picolines. *Magn Reson Chem* 47(3):228-238.

2-Ethoxy-3,4-dihzdro-3,5-dimethyl-2H-pyrane in DMSO

```
Current Data Parameters
NAME                 Ms1
EXPNO                  1
PROCNO                 1

F2 - Processing parameters
SI                 16384
SF           500.1377938 MHz
WDW                   no
SSB        0
LB         0 Hz
GB         0
PC                  1.00
```

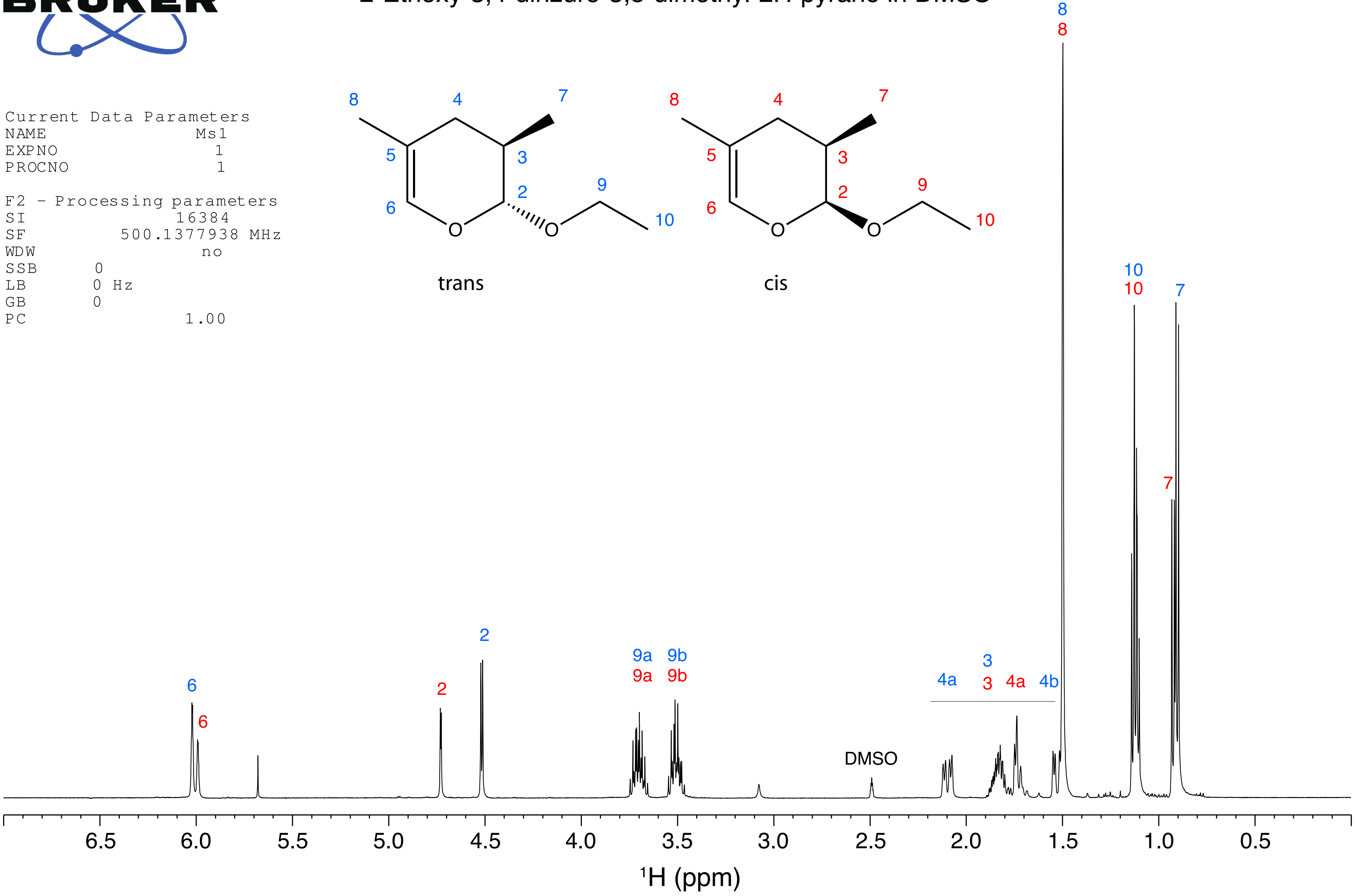

2-Ethoxy-3,4-dihzdro-3,5-dimethyl-2H-pyrane in DMSO

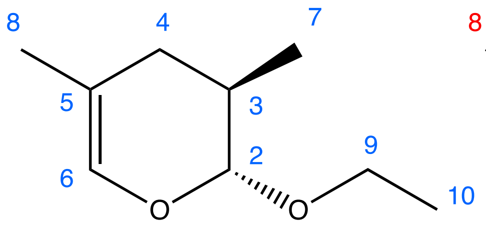

trans

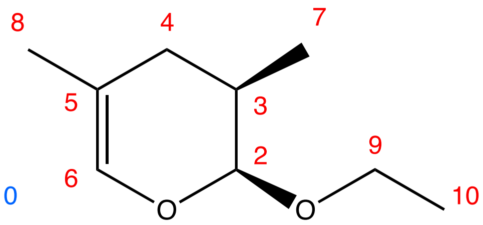

cis

```
Current Data Parameters
NAME                Ms0
EXPNO                 3
PROCNO                1

F2 - Processing paramet
SI                16384
SF           62.8962846 M
WDW                  no
SSB       0
LB                 1.20 H
GB        0
PC                 4.00
```

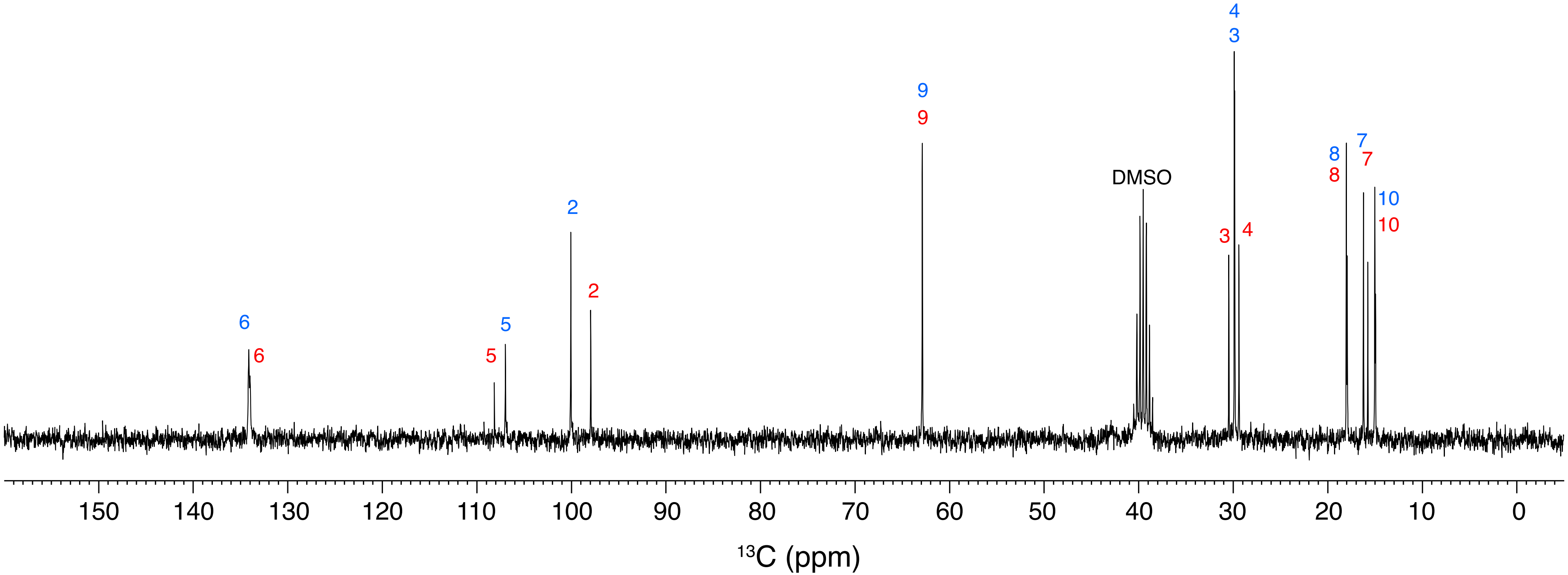

^{15}N-labeled 3,5-Dimethylpyridine in $CDCl_3$

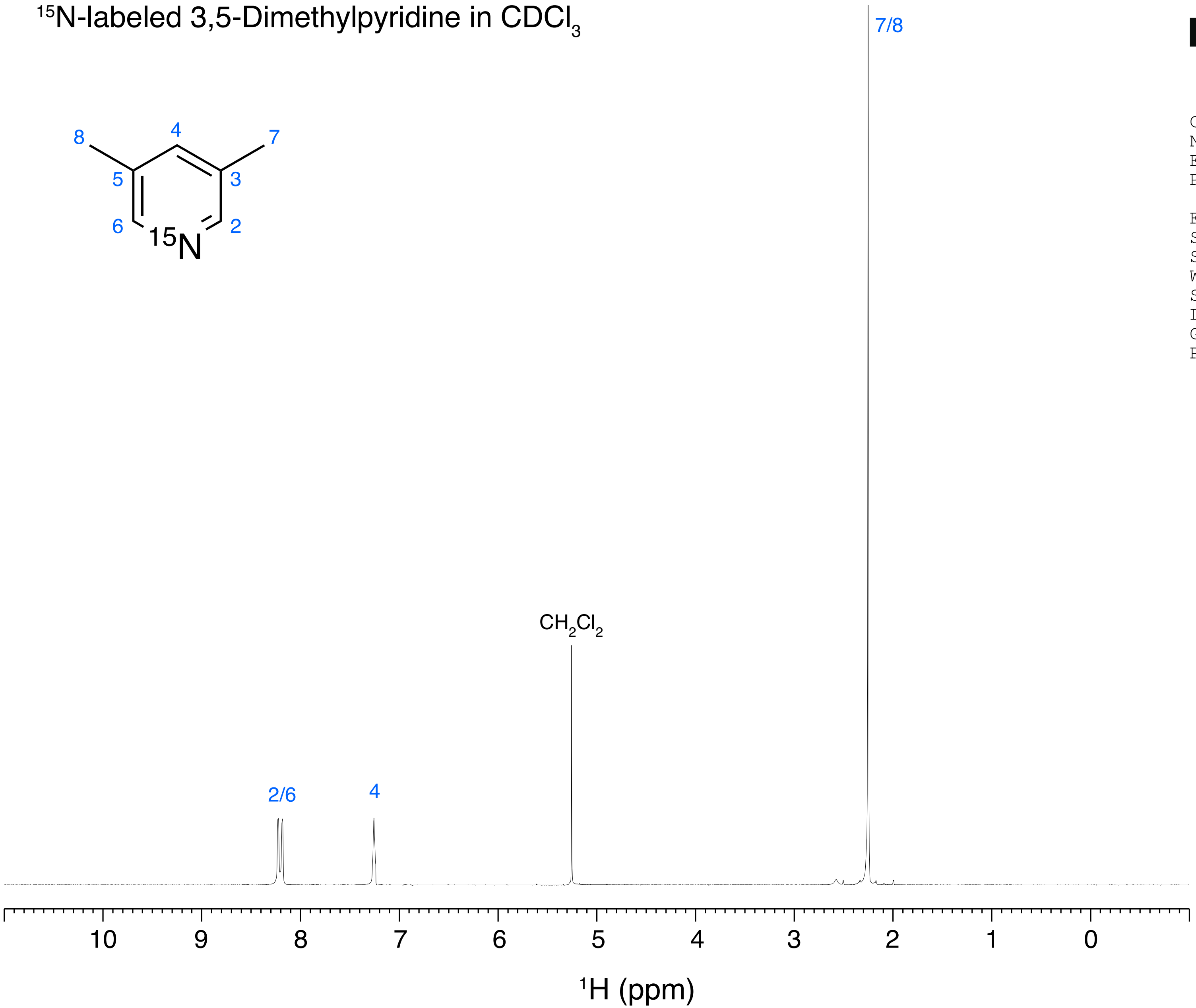

Current Data Parameters
NAME Ms0
EXPNO 5
PROCNO 1

F2 - Processing parameters
SI 8192
SF 250.1328590 MHz
WDW no
SSB 0
LB 0 Hz
GB 0
PC 4.00

^{15}N-labeled 3,5-Dimethylpyridine in $CDCl_3$

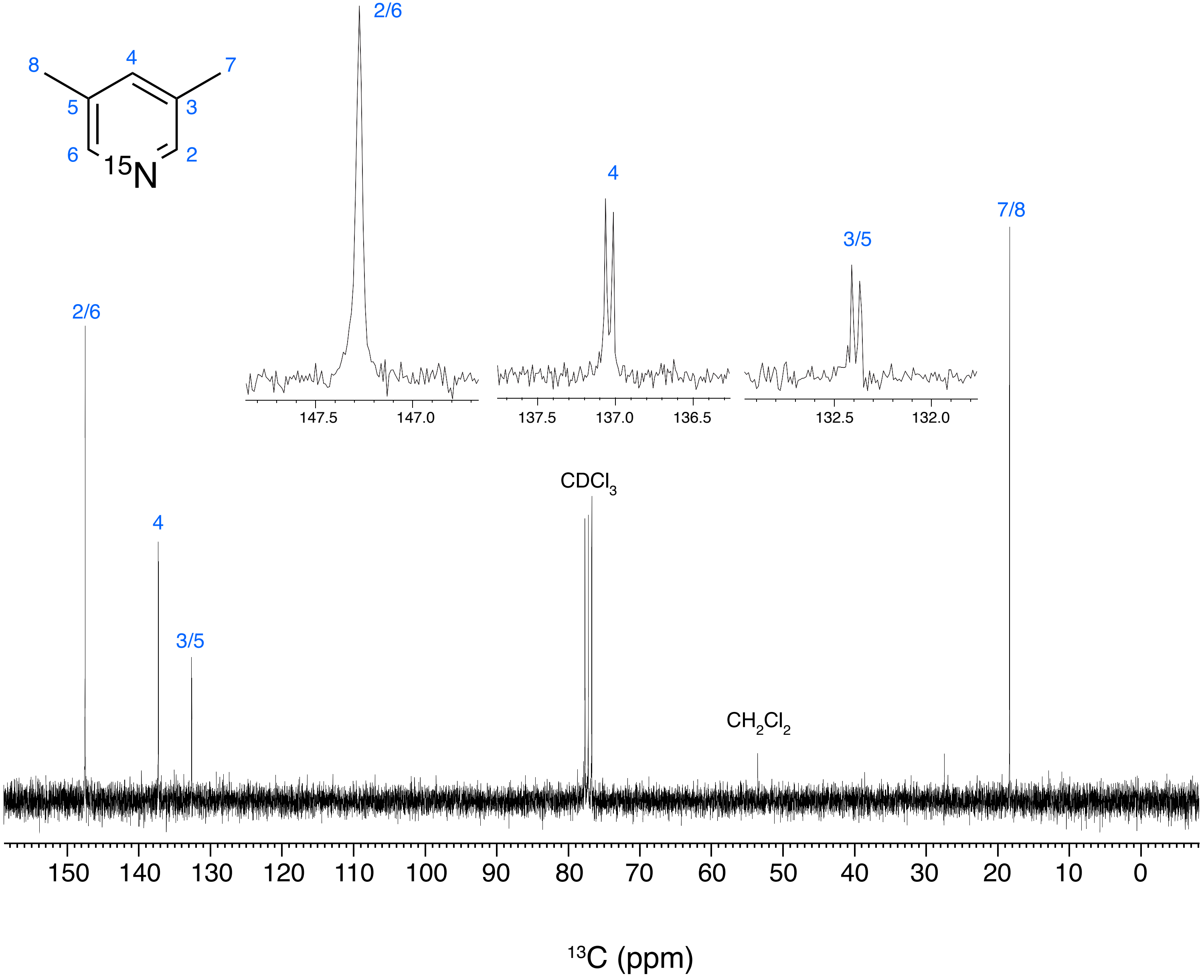


```
Current Data Parameters
NAME          MARIO13C_orig
EXPNO                     1
PROCNO                    1

F2 - Processing parameters
SI                    16384
SF               67.9249666 MHz
WDW                      no
SSB          0
LB                     2.00 Hz
GB           0
PC                     4.00
```

^{15}N-labeled 3,5-Dimethylpyridine in $CDCl_3$

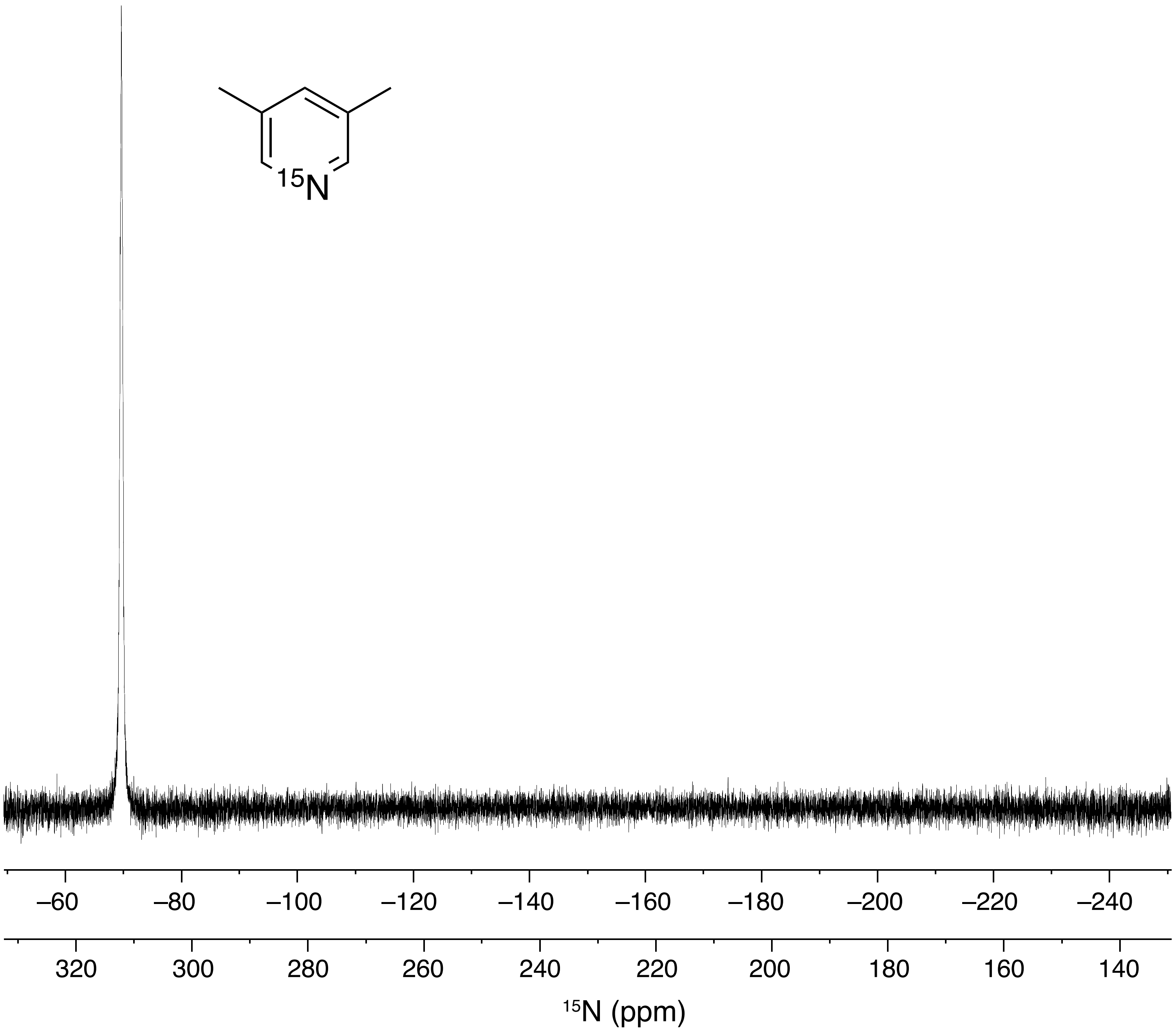

BRUKER

```
Current Data Parameters
NAME      ma_lut_15N_2018
EXPNO                   1
PROCNO                  1

F2 - Processing parameters
SI                  32768
SF             50.6976064 MHz
WDW                    no
SSB        0
LB         0  Hz
GB         0
PC                   1.00
```
